# Supplementary material for: Metabolomics Reveals Changes in Metabolite Profiles among Pre-Diapause, Diapause and Post-Diapause Larvae of Sitodiplosis mosellana (Diptera: Cecidomyiidae)
Source: Insects. 2022 Mar 30;13(4):339. doi: 10.3390/insects13040339 (PMC9032936; doi:10.3390/insects13040339)
Supplement: Supplementary file 1 [file insects-13-00339-s001.zip › insects-1623793-supplementary-new.pdf]

Table S1. Metabolites identified from pre-diapause, diapause and post-diapause larvae of *Sitodiplosis mosellana.*

Amino acids (29)	Sugars and polyols (22)	TCA intermediates (4)	Fatty acids (4)	Organic acids (16)	Others (19)
Proline	Trehalose	Fumaric acid	Oleic acid	Pantothenic acid	Phosphate
Alanine	Trehalose 6-phosphate	Malic acid	Linoleic acid	Lactic acid	Methyl phosphate
Threonine	Glucose	Citric acid	Palmitic acid	Oxalic acid	Glycerol- 1-phosphate
Leucine	Glucose-1-phosphate	α-Ketoglutaric acid	Stearic acid	Malonic acid	Inosine
Isoleucine	Fructose 1,6-bisphosphate			Galactonic acid	Inosine 5'-monophosphate
Valine	Maltose			Citramalic acid	α-Ecdysone
Glycine	Isomaltose			Phosphoglycolic acid	Putrescine
Serine	Lyxose			Phosphogluconic acid	β-Mannosylglycerate
Tryptophan	Lactose			3-Aminoisobutyric acid	Urea
Lysine	Tagatose			Hydroxyphenylacetic acid	Uridine
Phenylalanine	Lactulose			Aminooxyacetic acid	Uracil
Tyrosine	Talose			Aminomalonic acid	Uric acid
Methionine	Ribose 5-phosphate			2,4-Diaminobutyric acid	Glycocyamine
Cystine	Ribulose 5-phosphate			Dehydroascorbic acid	Hydroxylamine
Asparagine	Glycerol			Pentadecanoic acid	Adenosine 5-monophosphate
Glutamine	Sorbitol			Pyruvic acid	2-Hydroxypyridine
β-Alanine	Mannitol				7-α-Hydroxycholesterol
Glutamic acid	myo-Inositol				β-Glycerophosphoric acid
Ornithine	Xylitol				Cholecalciferol
Citrulline	Galactinol				
Oxoproline	Palatinitol				
Norleucine	Diglycerol				
Cysteine					
Homoserine					
Allothreonine					
Phosphoserine					
N-acetyl-L-leucine					
3-Hydroxy-L-proline					
4-Aminobutyric acid					

Table S2. Significantly different metabolites in diapause (D), post-diapause quiescence (PDQ) and developing (PDD) larvae of *Sitosiplosis mosellnana* compared to pre-diapause (PreD).

**Metabolites**	**D/PreD**			**PDQ/PreD**			**PDD/PreD**		
	**FC**	***P***	**VIP**	**FC**	***P***	**VIP**	**FC**	***P***	**VIP**
Amino acids (20)									
Alanine	2.28	<0.001	1.32	2.55	<0.001	1.29			
Proline	2.00	<0.001	1.83	4.82	<0.001	1.39			
4-Aminobutyric acid (GABA)	0.06	<0.001	1.33	0.09	<0.001	1.25	0.10	<0.001	1.28
Cysteine	0.07	<0.001	1.35	0.13	<0.001	1.27	0.17	<0.001	1.31
Asparagine	0.08	<0.001	1.31	0.41	<0.001	1.10			
Threonine	0.08	<0.001	1.33	0.10	<0.001	1.24	0.38	<0.001	1.25
Glycine	0.09	<0.001	1.35	0.11	<0.001	1.28	0.49	<0.001	1.18
Phenylalanine	0.13	<0.001	1.33	0.22	<0.001	1.21			
Leucine	0.20	<0.001	1.34	0.25	<0.001	1.19			
Serine	0.22	<0.001	1.33	0.26	<0.001	1.24			
Isoleucine	0.31	<0.001	1.32	0.29	<0.001	1.20			
Methionine	0.34	0.002	1.20	0.35	0.002	1.13			
β-Alanine	0.37	<0.001	1.44	2.52	<0.001	1.67	2.52	<0.001	1.39
Glutamine	0.38	<0.001	1.36	2.88	<0.001	1.59	0.38	<0.001	1.42
Allothreonine	0.39	<0.001	1.31	0.38	<0.001	1.16			
Glutamic acid				0.38	0.003	1.07	0.36	0.002	1.13
Tryptophan							3.52	<0.001	1.33
Cystine							3.33	<0.001	1.23
Ornithine							3.28	<0.001	1.16
Lysine							2.58	<0.001	1.23
Sugars and polyols (19)									
Trehalose 6-phosphate	321.53	<0.001	1.48	298.06	<0.001	1.63	144.52	<0.001	1.65
Trehalose	4.65	<0.001	1.65	11.56	<0.001	1.44	2.16	<0.001	1.73
Ribulose 5-phosphate	2.35	<0.001	1.46	4.95	<0.001	1.11	10.74	0.006	1.35
Talose	0.03	<0.001	1.36	0.06	<0.001	1.25	0.30	<0.001	1.29
Maltose	0.05	<0.001	1.29	0.04	<0.001	1.23	0.38	0.012	1.06
Isomaltose	0.06	<0.001	1.30	0.07	<0.001	1.20	0.19	<0.001	1.22
Lyxose	0.13	<0.001	1.35	0.13	<0.001	1.28	0.33	<0.001	1.30
Tagatose	0.13	<0.001	1.33	0.20	<0.001	1.23	0.29	<0.001	1.26
Fructose 1,6-bisphosphate	0.16	<0.001	1.33	0.18	<0.001	1.27	0.38	<0.001	1.26

Table S2. *Continued.*

Metabolites	D/PreD			PDQ/PreD			PDD/PreD		
	FC	*P*	VIP	FC	*P*	VIP	FC	*P*	VIP
Lactose	0.25	<0.001	1.33	0.33	<0.001	1.25	0.37	<0.001	1.27
Glucose-1-phosphate	0.35	<0.001	1.33	0.38	<0.001	1.25			
Glucose	0.37	<0.001	1.33	0.38	<0.001	1.23			
Mannitol	56.45	<0.001	1.49	50.25	<0.001	1.88	9.84	<0.001	1.67
Glycerol	10.50	<0.001	1.31	12.31	<0.001	1.48	4.54	<0.001	1.32
Palatinitol	3.19	<0.001	1.07	3.52	<0.001	1.08			
Galactinol	0.03	<0.001	1.31	0.02	<0.001	1.25	0.06	<0.001	1.25
Xylitol	0.04	<0.001	1.33	0.06	<0.001	1.26	0.36	<0.001	1.20
Myo-inositol	0.40	<0.001	1.14	0.30	0.002	1.17	0.05	<0.001	1.30
Diglycerol	0.33	<0.001	1.24	0.33	0.002	1.03			
TCA intermediates (4)									
α-Ketoglutaric acid	0.03	<0.001	1.40	0.46	<0.001	1.92	2.01	<0.001	1.58
Fumaric acid	0.19	<0.001	1.58	0.01	<0.001	1.41	0.39	<0.001	1.78
Citric acid	0.38	<0.001	1.73	0.45	<0.001	1.68			
Malic acid	0.48	<0.001	1.41				0.19	<0.001	1.43
Fatty acids (4)									
Linoleic acid	3.91	0.006	1.43	4.46	0.005	1.42			
Oleic acid	4.40	<0.001	1.32	4.84	<0.001	1.43			
Stearic acid	0.17	<0.001	1.24	0.16	<0.001	1.37	0.49	<0.001	1.23
Palmitic acid	0.18	<0.001	1.37	0.14	<0.001	1.50			
Organic acids (5)									
Pyruvic acid	3.17	<0.001	1.36	8.75	<0.001	1.43	8.69	<0.001	1.61
Pantothenic acid	4.86	<0.001	1.28	4.40	<0.001	1.26	4.85	<0.001	1.30
Galactonic acid	0.01	<0.001	1.35	0.01	<0.001	1.28	0.03	<0.001	1.31
Lactic acid	0.13	<0.001	1.33	0.15	<0.001	1.40	0.37	<0.001	1.39
6-Phosphogluconic acid	0.16	<0.001	1.30	0.46	<0.001	1.28			
Others (2)									
Uracil	0.39	<0.001	1.16	0.25	<0.001	1.29	0.13	<0.001	1.31
Urea	0.23	0.002	1.18	0.21	<0.001	1.23			

The fold change (FC) value was based on comparison of the means of peak intensity of six replicates between two groups. *P* values are obtained from student's t-test. VIP is variable importance in the projection of OPLS-DA model. The same for Table S3.

Table S3. Significantly different metabolites of pairwise comparisons between diapause (D), post-diapause quiescence (PDQ) and developing larvae (PDD) of *Sitosiplosis mosellnana.*

**Metabolites**	**PDQ/D**			**PDD/D**			**PDD/PDQ**		
	**FC**	***P***	**VIP**	**Fold**	***P***	**VIP**	**Fold**	***P***	**VIP**
Amino acids (18)									
Glutamine	7.47	<0.001	1.59				0.13	<0.001	1.56
β-Alanine	6.84	<0.001	1.42	6.88	<0.001	1.64			
Asparagine	5.25	<0.001	1.22	13.75	<0.001	1.28	2.62	<0.001	1.13
Proline	2.41	<0.001	1.67				0.23	<0.001	1.32
Phenylalanine				6.62	<0.001	1.29	3.86	<0.001	1.21
Glycine				5.25	<0.001	1.30	4.52	<0.001	1.24
Ornithine				4.97	<0.001	1.14	5.82	<0.001	1.13
Threonine				4.82	<0.001	1.13	3.80	<0.001	1.15
Leucine				4.60	<0.001	1.28	3.65	<0.001	1.17
Serine				3.48	<0.001	1.28	2.89	<0.001	1.22
Lysine				3.42	<0.001	1.24	2.60	<0.001	1.15
Tryptophan				3.28	<0.001	1.29	2.82	<0.001	1.10
Isoleucine				2.89	<0.001	1.27	3.11	<0.001	1.15
Methionine				2.61	<0.001	1.17	2.58	<0.001	1.23
Cystine				2.46	<0.001	1.14	2.16	<0.001	1.07
Cysteine				2.33	<0.001	1.18			
Allothreonine				2.00	<0.001	1.08	2.04	<0.001	1.03
Alanine				0.48	<0.001	1.32	0.43	<0.001	1.25
Sugars and polyols (18)									
Trehalose	2.48	<0.001	1.53	0.46	<0.001	1.35	0.19	<0.001	1.35
Ribulose 5-phosphate	2.11	<0.001	1.23	4.58	<0.001	1.12	2.17	<0.001	1.22
Talose				9.19	<0.001	1.31	5.03	<0.001	1.15
Maltose				7.02	<0.001	1.23	8.97	<0.001	1.18
Isomaltose				3.32	<0.001	1.13	2.71	<0.001	1.06
Glucose				2.78	<0.001	1.23	2.70	<0.001	1.24
Glucose 1-phosphate				2.74	<0.001	1.25	2.53	<0.001	1.13
Lyxose				2.62	<0.001	1.26	2.61	<0.001	1.15
Fructose 1,6-bisphosphate				2.41	<0.001	1.25	2.17	<0.001	1.24
Xylitol				9.66	<0.001	1.28	5.82	<0.001	1.20

Table S3. *Continued.*

Metabolites	PDQ/D			PDD/D			PDD/PDQ		
	FC	*P*	VIP	Fold	*P*	VIP	Fold	*P*	VIP
Diglycerol				2.79	<0.001	1.28	2.75	<0.001	1.20
Galactinol				2.40	<0.001	1.30	2.74	<0.001	1.32
Tagatose				2.30	<0.001	1.23			
Myo-inositol				0.13	<0.001	1.42	0.17	<0.001	1.39
Mannitol				0.17	<0.001	1.59	0.20	<0.001	1.67
Palatinitol				0.32	<0.001	1.28	0.29	<0.001	1.17
Glycerol				0.43	<0.001	1.27	0.37	<0.001	1.63
Trehalose 6-phosphate				0.45	<0.001	1.73	0.48	<0.001	1.30
TCA intermediates (4)									
α-Ketoglutaric acid	14.20	<0.001	1.42	61.90	<0.001	1.38	4.36	<0.001	1.61
Fumaric acid	0.05	<0.001	1.43	2.07	<0.001	1.32	40.73	<0.001	1.28
Citric acid				2.92	<0.001	1.63	2.48	<0.001	1.47
Malic acid				2.22	<0.001	1.39	2.19	<0.001	1.53
Fatty acids (4)									
Palmitic acid				3.99	<0.001	1.64	4.90	<0.001	1.33
Stearic acid				2.89	<0.001	1.38	3.14	<0.001	1.52
Oleic acid				0.16	<0.001	1.41	0.15	<0.001	1.65
Linoleic acid				0.24	<0.001	1.57	0.21	<0.001	1.59
Organic acids (4)									
6-Phosphogluconic acid	2.92	<0.001	1.14	6.10	<0.001	1.23	2.09	<0.001	1.20
Galactonic acid				4.49	<0.001	1.15	2.35	<0.001	1.22
Lactic acid				2.88	<0.001	1.22	2.57	<0.001	1.43
Pyruvic acid	2.76	<0.001	1.56	2.74	<0.001	1.58			
Others (2)									
Urea				2.69	<0.001	1.32	2.80	<0.001	1.19
Uracil				0.33	<0.001	1.20			

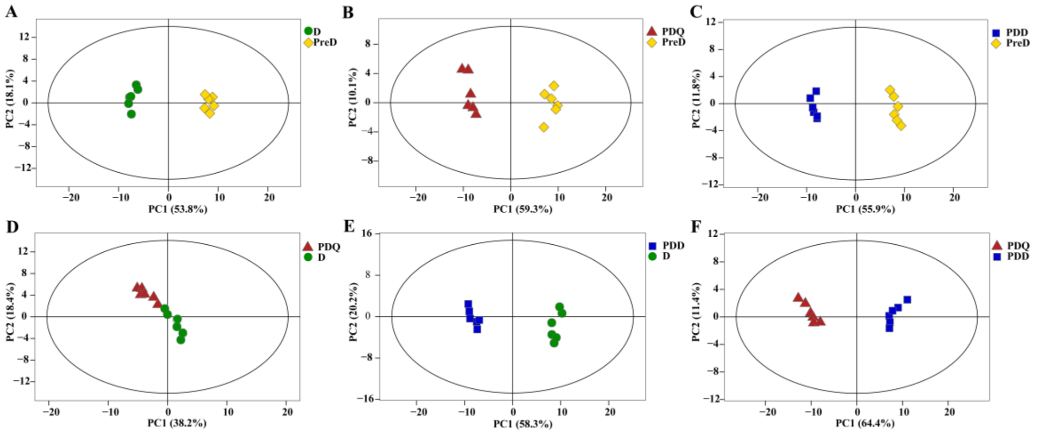

Figure S1. Principal component analysis (PCA) score plots of the metabolic profiles of pair comparisons of the four groups, including pre-diapause (PreD), diapause (D), post-diapause quiescence (PDQ) and post-diapause developmental larvae (PDD) of *Sitodiplosis mosellana*.
